# Dr. Walter Scott Hicks

**Published:** 1914-05

**Authors:** 


**Dr. Walter Scott Hicks,** Buffalo 1851, died at his home in Bristol March 16, aged 86.

